# Extracellular Vesicles: Biology and Therapeutic Applications

**DOI:** 10.3390/ijms252313034

**Published:** 2024-12-04

**Authors:** Tamás Visnovitz

**Affiliations:** Department of Genetics, Cell- and Immunobiology, Semmelweis University, Nagyvárad tér 4, 1089 Budapest, Hungary; visnovitz.tamas@semmelweis.hu

## 1. Introduction

Extracellular vesicles (EVs) are phospholipid-bilayer-limited particles in the extracellular space, without self-replicating capabilities [1,2]. They represent a heterogeneous population in terms of size and origin, playing pivotal roles in both physiological and pathological processes [3]. The EV-related research field has been exponentially growing over the past two decades, which demonstrates its impact [4]; however, many EV-related questions remain unanswered [3].

Up to a few years ago, only three subtypes of EVs (exosomes, microvesicles, and apoptotic bodies) were known [5]. Recently, however, several new EV types have been identified based on their mechanisms of release and origin [6]. According to the latest MISEV2023 guidelines, EVs are categorized into two main subtypes based on their biogenesis: exosomes and ectosomes [2]; however, recent studies have highlighted a novel and general mechanism for small EV secretion, where ectocytosis plays a crucial role in exosome release. In this process, large multivesicular structures bud from the plasma membrane via ectocytosis, and intraluminal vesicles of endomembrane origin are released into the extracellular space through a “torn bag mechanism” [7,8].

Despite the growing amount of knowledge and promising translational applications of EVs as biomarkers and therapeutics [9], many aspects of their biology remain unclear. The exact roles of various EV populations in intercellular communication, their release mechanisms, uptake pathways, and intracellular fates are not yet fully understood. High-throughput methods have uncovered numerous EV-related biomarkers, paving the way for novel diagnostic applications, although the clinical validation of these markers remains incomplete. Additionally, EV-based therapies are an emerging and rapidly growing field within biomedical science.

The EV research community continuously faces significant technical and standardization challenges [3]. Considerable efforts have been made to address these issues. The International Society for Extracellular Vesicles (ISEV) has released successive guidelines such as MISEV2014, MISEV2018, and MISEV2023 [1,2,10]. However, the technologies employed in EV research are still evolving, and further developments are essential.

This Special Issue in *IJMS* aims at presenting a collection of research and review papers on EV-related biological processes, technological advancements, biomarker discovery, and therapeutic applications.

## 2. New Results

The research articles featured here provide detailed experimental findings and explore the roles of EVs in various contexts, including reproductive biology, disease mechanisms, and regenerative medicine.

In reproductive biology, EVs are crucial in early embryo–maternal interactions and oocyte maturation. For instance, a study on bovine embryos revealed that EVs secreted during blastulation modulate gene expression in bovine endometrial cells. Notably, embryos produced in vitro induced twice as many differentially expressed genes in endometrial cells as those produced in vivo, indicating that the origin of embryos may determine which pathways are activated in endometrial cells [11]. Another study demonstrated that follicular fluid-derived EVs enhance cumulus cell expansion, promoting viability and transcriptomic changes during oocyte maturation in equine reproductive systems [12]. These findings highlight the importance of EVs in reproductive processes and their potential applications in in vitro fertilization. EV-derived miRNAs also offer insights into developmental biology and infant health. miRNAs delivered via maternal plasma and milk EVs influence critical developmental processes. Factors such as delivery mode and maternal BMI significantly affect EV composition, highlighting the need to account for these variables in future research [13].

The majority of studies in this Special Issue focus on the link between EVs and various diseases. EVs act as “Janus-faced” particles, contributing to disease mechanisms while offering therapeutic potential. In HIV-positive individuals, plasma EVs are enriched with miRNAs such as let-7b-5p, which may help explain the increased cerebrocardiovascular risks in this population [14]. In antiphospholipid syndrome, EVs carry specific miRNAs (miR-483-3p and miR-326) that promote endothelial cell proliferation, activate adhesion molecules/procoagulant factors and monocytes, and contribute to thrombosis and inflammation. These miRNAs may serve as biomarkers for disease severity and therapeutic targets [15]. Osteoblast-derived EVs, taken up via caveola-dependent endocytosis, regulate bone remodeling and immune responses. They carry miRNAs that influence osteogenesis and M2 macrophage polarization, contributing to skeletal homeostasis [16].

In cancer research, EVs are emerging as powerful tools for targeted therapy. Engineered EVs can deliver therapeutic cargo, including anti-cancer agents, to refractory tumors. Compound-induced trafficking systems enable precise delivery to refractory tumors such as triple-negative breast cancer and pancreatic cancer [17]. Additionally, EVs derived from cancer cells themselves provide insights into tumor biology. For instance, in Ewing sarcoma, EVs carrying CD99 markers influence cell dissemination, differentiation, and death with potential applications in diagnostics and treatment [18].

EVs may also offer therapeutic applications. The released free heme in malaria and sickle cell anemia triggers inflammatory responses, aggravating tissue damage. EVs loaded with anti-inflammatory miRNAs, such as miR-451a and let-7i-5p, mitigate these effects by modulating pathways involved in inflammation. The applied technology may modulate heme-induced inflammation, paving the way for novel treatments for hemolytic disorders [19]. Moreover, EVs may be used in regenerative medicine. Mesenchymal stem cell-derived EVs have demonstrated remarkable efficacy in spinal cord injury recovery. In preclinical models, these EVs promote axonal remyelination and improve the reperfusion of damaged nervous tissue, leading to the partial restoration of locomotor activity [20].

Finally, a novel EV isolation technique described here enables EV purification directly from hepatic and adipose tissue samples, allowing studies in an in vivo microenvironment [21].

## 3. Reviews

The review articles in this Special Issue provide a broader perspective on the roles of EVs, summarizing recent advancements and identifying challenges in their application as therapeutic and diagnostic tools. They highlight the multiple applications of EVs and their potential in clinical translation.

In therapeutic applications, EVs have received increasing attention as drug delivery platforms due to their natural ability to protect cargo from degradation and target specific tissues. For instance, they can deliver gene-editing tools such as CRISPR/Cas9 and cancer therapeutics [22]. Understanding the mechanisms by which EVs target different organs and organ systems is essential for their therapeutic applications as drug delivery platforms. This knowledge can help determine optimal dosages and administration routes [23]. The bioengineering of EVs may facilitate the delivery of small molecules, RNA therapeutics, and proteins, particularly to modulate Th17-cell-dependent pathways. However, challenges related to EV production, cargo loading, and targeted delivery still persist. EV-based therapies hold great potential to revolutionize treatment strategies for Th17-cell-mediated diseases and establish EVs as tools in clinical applications [24].

In diagnostics, EVs may serve as non-invasive biomarkers for various diseases. For example, a review here shows that in non-alcoholic fatty liver disease, potential biomarker candidates can be identified using a multi-step approach for analyzing the scientific literature. The ability to analyze the miRNA and protein content of EVs enables the identification of disease-specific signatures through diagnostic tests, making them valuable tools in the future of medicine [25]. EVs also have promising roles in neuroregeneration. Stem cell-derived EVs facilitate nerve repair and recovery and may help in treating neurodegenerative diseases and peripheral nerve injuries [26]. Salivary EVs hold significant potential in biomarker analysis in ocular disease due to their accessibility, non-invasive collection, and stability. Specifically, for ocular diseases, salivary EVs may serve as a future diagnostic and therapeutic platform [27].

## 4. Conclusions

The studies presented in this Special Issue draw attention to the multifaceted roles of EVs in biology and medicine. They underscore their potential as diagnostic biomarkers, therapeutic agents, and drug delivery platforms. EVs are involved in diverse physiological and pathological contexts, including reproductive biology, oncology, regenerative medicine, and inflammatory diseases. While significant progress has been made, challenges such as standardization, efficient cargo loading, and targeted delivery persist. The progress detailed in this Special Issue paves the way for a deeper understanding and broader utilization of EVs in modern medicine.
